# Supplementary material for: Construction of a Glycaemia-Based Signature for Predicting Acute Kidney Injury in Ischaemic Stroke Patients after Endovascular Treatment
Source: J Clin Med. 2022 Jul 3;11(13):3865. doi: 10.3390/jcm11133865 (PMC9267863; doi:10.3390/jcm11133865)
Supplement: Supplementary file 1 [file jcm-11-03865-s001.zip › jcm-1768277-supplementary.pdf]

Table S1 Unadjusted and adjusted ORs of glycaemia in patients with and without DM

	Patients with DM (n=230)				Patients without DM (n=487)				P-interaction
	Crude OR (95% CI)	P	Adjusted OR (95% CI)	P	Crude OR (95% CI)	P	Adjusted OR (95% CI)	P	DM and glycemia
Acute glycemia	1.006(1.001-1.011)	0.019	1.005(0.999-1.011)	0.102	1.021(1.014-1.028)	<0.001	1.013(1.006-1.021)	<0.001	0.184
Chronic glycemia	1.000(0.995-1.006)	0.926			1.001(0.989-1.013)	0.886			0.685
A/C glycemic ratio	2.700(1.146-6.366)	0.023	2.568(0.907-7.273)	0.076	18.135(7.539-43.622)	<0.001	7.880(3.007-20.653)	<0.001	0.202
Δ_{A-C}	1.005(1.000-1.010)	0.041	1.004(0.999-1.010)	0.134	1.024(1.017-1.032)	<0.001	1.017(1.009-1.025)	<0.001	0.052

Adjusted for age, hypertension, atrial fibrillation, baseline NIHSS score, stroke subtype, ASITN/SIR, intravenous thrombolysis, number of devices passed and mTICI score

Abbreviations: AKI, acute kidney injury; OR, odds ratio; DM, diabetes mellitus; A/C, acute/chronic; Δ_{A-C}, the difference between acute and chronic glycaemia

Table S2 Receiver operating characteristic of glycaemia to predict AKI

	Area under the curve	95% confidence interval	P
Acute glycaemia	0.647	0.604-0.690	<0.001
Chronic glycaemia	0.518	0.472-0.565	0.440
A/C glycaemic ratio	0.657	0.612-0.702	<0.001
Δ A-C	0.650	0.604-0.696	<0.001

Abbreviations: AKI, acute kidney injury; A/C, acute/chronic; Δ_{A-C}, the difference between acute and chronic glycaemia
